# Evaluation of diagnostic techniques for early detection of heartworm in experimentally infected dogs: identification of *Dirofilaria immitis*-derived microRNA in the initial 28 weeks post-inoculation

**DOI:** 10.1186/s13071-024-06337-y

**Published:** 2024-06-13

**Authors:** Daniel Felipe Barrantes Murillo, Elyssa J. Campbell, Andrew R. Moorhead, Chengming Wang

**Affiliations:** 1 Department of Pathobiology, College of Veterinary Medicine, Auburn University, Auburn, AL USA; 2 Department of Infectious Veterinary Medicine, University of Georgia, Athens, GA USA

**Keywords:** *Dirofilaria immitis*, Antigen test, Heat treatment, PCR, Thick smear, miRNA, Heartworm

## Abstract

**Background:**

*Dirofilaria immitis,* commonly known as heartworm (HW), is a parasitic nematode transmitted by various mosquito species, leading to heartworm disease (HWD) in dogs. Diagnosis of HW typically involves antigen or microfilariae detection, or visualization of adult worms through imaging or post mortem examination. Polymerase chain reaction (PCR) and micro RNA (miRNA) detection have been explored for HW diagnosis.

**Methods:**

Three dogs, previously experimentally infected with HW, underwent blood sampling every 4 weeks for 7 months. Samples were assessed for antigen presence after heat treatment, PCR amplification, and microfilaria examination using Giemsa-stained thick smears. Additionally, whole blood aliquots underwent miRNA deep sequencing and bioinformatic analysis.

**Results:**

Heartworm antigen was detectable after heat treatment at 20 weeks post-inoculation and via PCR at 24 weeks, with microfilariae observed in peripheral blood smears at 28 weeks. However, deep miRNA sequencing revealed that the miRNA candidate sequences are not consistently expressed before 28 weeks of infection.

**Conclusions:**

While ancillary molecular methods such as PCR and miRNA sequencing may be less effective than antigen detection for detecting immature larval stages in an early stage of infection, our experimental findings demonstrate that circulating miRNAs can still be detected in 28 weeks post-infection.

**Graphical Abstract:**

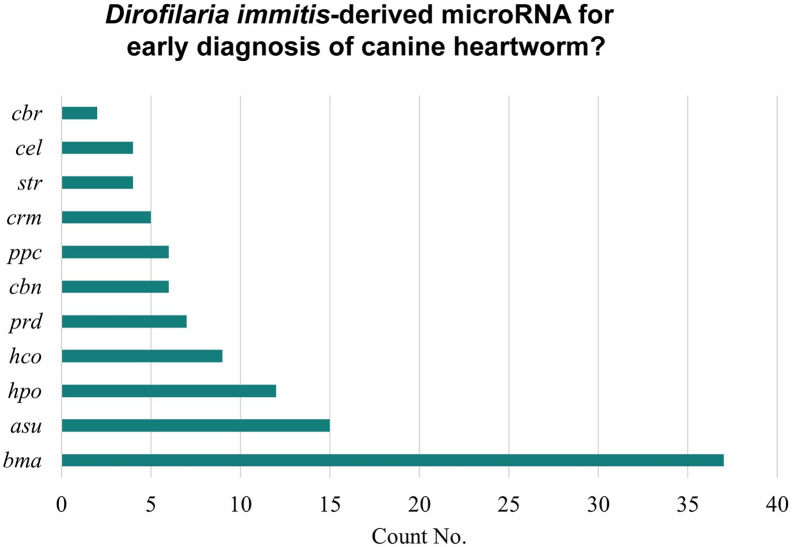

**Supplementary Information:**

The online version contains supplementary material available at 10.1186/s13071-024-06337-y.

## Background

*Dirofilaria immitis*, a nematode transmitted by several species of mosquito vectors belonging to the genera *Aedes, Anopheles*, and *Culex*, is the causing agent of heartworm disease (HWD) in dogs [1, 2]. While some infected animals may remain asymptomatic, the presence of immature and adult worms within the pulmonary vasculature often leads to clinical signs, exacerbated by physical activity [3]. Clinical manifestations of HWD include coughing, tachypnea, dyspnea, right-sided heart murmurs, jugular distension, syncope, ascites, poor body condition, and even sudden death [3, 4]. Chronic eosinophilic inflammation, villous endarteritis, vasculitis, and thrombosis are attributed to the presence of immature and adult worms within the vascular lumen of the pulmonary arteries [4]. The vascular lesions caused by parasites can lead to pulmonary hypertension and congestive heart failure [4].

In recent years, extensive research has focused on improving the diagnostic accuracy of HWD, consistently identifying the infection status as early as possible. Early detection of HWD is crucial to initiate treatment before the development of detrimental pathological lesions in the pulmonary arteries. Currently, diagnosis relies on various techniques including antigen detection in blood, serum, or plasma; microfilaremia assessment; or direct visualization of adult worms using imaging techniques or post mortem examination. However, these aforementioned diagnostic assays convey several pitfalls. The detection of antigens derived from the reproductive tract of adult female worms remains the most accurate test for heartworm (HW) diagnosis, yet its sensitivity may be influenced by worm burden, single-sex infections, antigenemia levels, and host immune responses [4–6], with antigen–antibody complexes potentially yielding false-negative results [6] and cross-reactions with other dirofilarial and nematode species, inducing false-positive results [5, 7, 8].

To address this challenge in HW diagnosis, additional molecular methods have emerged, primarily for experimental and research purposes. Polymerase chain reaction (PCR), a highly sensitive molecular diagnostic technique for *D. immitis*, enables species differentiation within the same genus [9–13]. Studies have also identified *D. immitis*-derived microRNAs (miRNAs) as potential early infection markers [14–18].

To explore the presence of circulating *D. immitis* miRNAs and their potential application in HW infection detection, we conducted a comparative study in experimentally infected dogs (*Canis lupus familiaris*), collecting blood samples at 28-day intervals over 7 months. We hypothesized that infective, developmental larval stages and immature worms may be detectable through secreted molecular markers in peripheral blood before the release of antigen upon arrival in the pulmonary arteries. These molecular markers will be consistently detected in blood and used as potential biomarkers for HW diagnosis. Concurrently, PCR, antigen testing after heat treatment, and microfilaria examination via Giemsa-stained thick blood smears were performed on each sample to evaluate diagnostic efficacy.

## Methods

### Canine plasma samples

The samples utilized in this study were obtained from purpose-bred dogs, experimentally infected with *D. immitis* by the Department of Infectious Diseases, College of Veterinary Medicine, University of Georgia, USA. Three dogs, identified as Animals ID: “I”, “J”, and “M”, were inoculated via inguinal subcutaneous injection of 50 infective third-stage larvae (L3) of the Missouri strain of *D. immitis*, following a previously published inoculation protocol [19].

A total of 2 mL of whole blood was collected from each dog in an ethylenediaminetetraacetic acid (EDTA) tube on 21 November 2022, before HW inoculation, identified as I0, J0, and M0, respectively, and used as a negative control in all assays. Serial blood samples (2 mL) were then taken from each dog at 28-day intervals for 7 months corresponding to 4, 8, 12, 16, 20, 24, and 28 weeks post-infection, and identified as follows: I1, I2, I3, I4, I5, I6, I7, J1, J2, J3, J4, J5, J6, J7, M1, M2, M3, M4, M5, M6, and M7. Following collection, the samples were centrifuged at 650 × *g* for 15 min to separate plasma from cellular components of the blood and stored at −80 °C. An aliquot of 400 µL of the centrifuged cellular components of the blood was used for PCR, while a whole blood aliquot of approximately 600–900 µL was used for RNA extraction and sequencing. Additional blood (1 mL) was used for antigen testing and quantification of microfilaria using Giemsa stain on thick blood smears (60 µl) by triplicates (20 µl per slide).

All sample collection and experimental procedures were conducted following the guidelines of the Institutional Animal Care and Use Committee at the University of Georgia (Protocol A2019-04).

### Heat treatment and antigen detection

Each blood sample underwent heat treatment before antigen detection. Briefly, 2 mL of blood contained within an EDTA tube was centrifuged at 650 × *g* for 20 min at 23 °C. The resulting supernatant plasma (1.0 ml) was transferred to a 2 mL microcentrifuge tube and incubated in a heat block at 104 °C for 10 min. Subsequently, an aliquot of 50 µL from each sample was evaluated using a well-based commercial antigen capture ELISA (DiroCHEK^®^ Heartworm Antigen Test Kit; Zoetis, Florham Park, NJ, USA) following the manufacturer’s instructions. Antigen testing was conducted visually to determine the presence or absence of antigen by observing the color change on the DiroCHEK^®^ as per the manufacturer’s instructions.

### Microfilariae quantification

The Giemsa-stained thick blood smears were prepared using a published protocol [19]. In brief, a total of 50 µL of water was pipetted onto the glass slide before adding 20 µL anticoagulated blood. The water and blood were mixed and spread over a 15 mm × 25 mm rectangular area using a toothpick and the slides were dried overnight at room temperature so the water could lyse the red blood cells. Once dried, the slides were placed on a slide warmer for 1 h at 50 °C to fix the sample to the slide. Subsequently, the slides were covered entirely with 10% Giemsa (VWR Scientific, Radnor, PA, USA) in Tris–acetate-EDTA (TAE) buffer for 10 min. After washing the excess stain with deionized water, the slides were air-dried and examined using a compound microscope (Nikon Eclipse E200, Nikon Corporation, Tokyo, Japan) at 400× magnification. Microfilariae were counted in a marked area using a zigzagging grid pattern, and the concentration was calculated using the formula

$${mf/mL}_{total}= {mf}_{sample} \text{x }50$$

where ${mf/mL}_{total}$ was the concentration of microfilariae per milliliter of whole blood and ${mf}_{sample}$ was the total number of microfilariae in a slide. The test was performed in triplicate and the average count was provided.

### DNA extraction and PCR detection of *D. immitis*

The DNA extraction protocol required 400 µL aliquots centrifuged blood samples without the plasma and was performed using the High-Pure PCR Template Preparation Kit (Roche Molecular Biochemicals, Indianapolis, IN, USA) according to the manufacturer’s instructions [9, 20]. The DNA was eluted in 50 µL elution buffer and preserved at −80 ℃ until utilized for PCR.

The quantitative PCR applied used the following primers: ImmF: 5′-CTA TAT GTT ACC TTA ATT GG-3′; ImmR: 5′-CTT AAC CAT TAT CTT AGA TCA G-3′; and the probe ImmT: 5′-ROX-GTA GCT AGT AAG TTT ACC TTG-BHQ2-3′; ROX = 6-carboxy-Xrhodamine, BHQ2 = black hole quencher 2; the amplicon size is 162 bp. Those primers target the 16S rRNA gene of *D. immitis* [9, 10]. Roche Light Cycler 480 II thermocycler (Roche Diagnostics GmbH, Mannheim, Germany) was used to perform the PCR. Briefly, 10 µL of the extracted DNA was added to a 10 µL reaction mixture containing 5 × PCR FRET buffer, 400 µM dNTP (Roche Diagnostics GmbH), 0.34 units of Platinum Taq DNA Polymerase (Invitrogen, Carlsbad, CA, USA), 1 µM of each forward and reverse primer (Integrated DNA Technologies, Coralville, IA, USA), and a final volume of molecular grade nuclease-free water [9]. The PCR amplicons obtained from DNA extracted from *D. immitis* positive samples provided by the Molecular Diagnostic Laboratory at Auburn University were used as a positive control.

### Analysis of miRNA from canine whole-blood

Total RNA including miRNA was extracted from dog whole blood using miRNeasy (QIAGEN, Germantown, MD, USA). All extracted RNA was used in the library preparation following Illumina’s TruSeq-small-RNA-sample preparation protocols (Illumina, San Diego, CA, USA). Quality control analysis and quantification of the DNA library were performed using Agilent Technologies 2100 Bioanalyzer High Sensitivity DNA Chip. Single-end sequencing 50 bp was performed on Illumina’s Hiseq 2500 sequencing system following the manufacturer’s recommended protocols.

Raw reads were subjected to an in-house program, ACGT101-miR (LC Sciences, Houston, Texas, USA) to remove adapter dimers, junk, low complexity, common RNA families (rRNA, tRNA, snRNA, snoRNA), and repeats. The reads were mapped to mature and precursor miRNA sequences of *C*. *familiaris* and other mammalian species available in miRbase 22.0. Subsequently, unique sequences with length in 18 ~ 26 nucleotide were mapped to all nematode species precursors in miRBase 22.0 (*Ascaris suum, Brugia malayi, Caenorhabditis brenneri, Caenorhabditis briggsae, Caenorhabditis elegans, Caenorhabditis remanei, Haemonchus contortus, Heligmosomoides polygyrus, Panagrellus redivivus, Pristionchus pacificus, Strongyloides ratti*) by BLAST search to identify known miRNAs and novel 3p- and 5p- derived miRNAs. Length variation at both 3’ and 5’ ends and one mismatch inside of the sequence were allowed in the alignment. The unique sequences mapping to specific species of mature miRNAs in hairpin arms were identified as known miRNAs and conserved *D. immitis* miRNAs. The unique sequences mapping to the other arm of known specific species precursor hairpin opposite to the annotated mature miRNA-containing arm were novel 5p- or 3p-derived miRNA candidates. The remaining sequences were mapped to other selected species precursors (with the exclusion of specific species) in miRBase 22.0 by BLAST search, and the mapped pre-miRNAs were further BLAST searched against the specific species genomes to determine their genomic locations. The above two we defined as known miRNAs.

The unmapped sequences were BLAST searched against the specific *D. immitis* genome (https://parasite.wormbase.org/Dirofilaria_immitis_prjeb1797/Info/Index/#:~:text=The%20D.,2.2%20from%20nematodes.org), and the hairpin RNA structures containing sequences were predicated from the flank 80 nt sequences using RNAfold software (http://rna.tbi.univie.ac.at/cgi-bin/RNAfold.cgi). The criteria for secondary structure prediction were: (1) number of nucleotides in one bulge in the stem (≤ 12), (2) number of base pairs in the stem region of the predicted hairpin (≥ 16), (3) cutoff of free energy (kCal/mol ≤ −15), (4) length of hairpin (up and down stems + terminal loop ≥ 50), (5) length of hairpin loop (≤ 20), (6) number of nucleotides in one bulge in mature region (≤ 8), (7) number of biased errors in one bulge in mature region (≤ 4), (8) number of biased bulges in mature region (≤ 2), (9) number of errors in mature region (≤ 7), (10) number of base pairs in the mature region of the predicted hairpin (≥ 12), and (11) percentage of mature in stem (≥ 80).

Differential expressions of miRNAs based on normalized deep-sequencing counts were analyzed using statistical tests such as the Fisher’s exact test, chi-squared 2 × 2 test, chi-squared n × n test, Student’s *t*-test, or analysis of variance (ANOVA), depending on the experimental design. A significant threshold of 0.01 or 0.05 was set for each test.

To predict genes targeted by the most abundant miRNAs, two computational target prediction algorithms (TargetScan 50 and Miranda 3.3a) were employed to identify miRNA binding sites. Predictions from both algorithms were combined, and overlaps were calculated. Additionally, Gene Ontology (GO) terms and Kyoto Encyclopedia of Genes and Genomes (KEGG) pathway annotations of the most abundant miRNAs and miRNA targets were performed to further elucidate their functional roles. This comprehensive approach enabled the identification of circulating miRNAs derived from *D. immitis* and provided insights into their potential roles as early diagnostic makers and regulators of host–parasite interactions.

## Results

Heat treatment before antigen testing allowed for HW detection 20 weeks post-infection in all three dogs. PCR detection of HW was achieved 24 weeks post-infection in all subjects, following DNA extraction using the cellular component of the blood (buffy coat and erythrocytes) without plasma removal after centrifugation. Microfilariae were visualized on Giemsa-stained thick blood smears 28 weeks post-infection in all three dogs (Table 1).

**Table 1:** Summary of heartworm diagnosis with four methods in this study

Subject	Diagnostic method	Weeks post-infection							
		0**	4	8	12	16	20	24	28
I	Ag test*	–	–	–	–	–	Pos	Pos	Pos
	Thick smear	–	–	–	–	–	–	–	100**
	PCR	–	–	–	–	–	–	Pos	Pos
	miRNA	–	–	–	–	–	–	–	Detected
J	Ag test	–	–	–	–	–	Pos	Pos	Pos
	Thick smear	–	–	–	–	–	–	–	16.67**
	PCR	–	–	–	–	–	–	Pos	Pos
	miRNA	–	–	–	–	–	–	–	Detected
M	Ag test	–	–	–	–	–	Pos	Pos	Pos
	Thick smear	–	–	–	–	–	–	–	33.33**
	PCR	–	–	–	–	–	–	+	Pos
	miRNA	–	–	–	–	–	–	–	Detected

^*^The Ag test was performed after the heat treatment. *The negative control used was a blood sample taken from each subject the day before inoculation with HW. **The results are reported as microfilaria per milliliter and correspond to the average of the triplicates

**Fig. 1:**
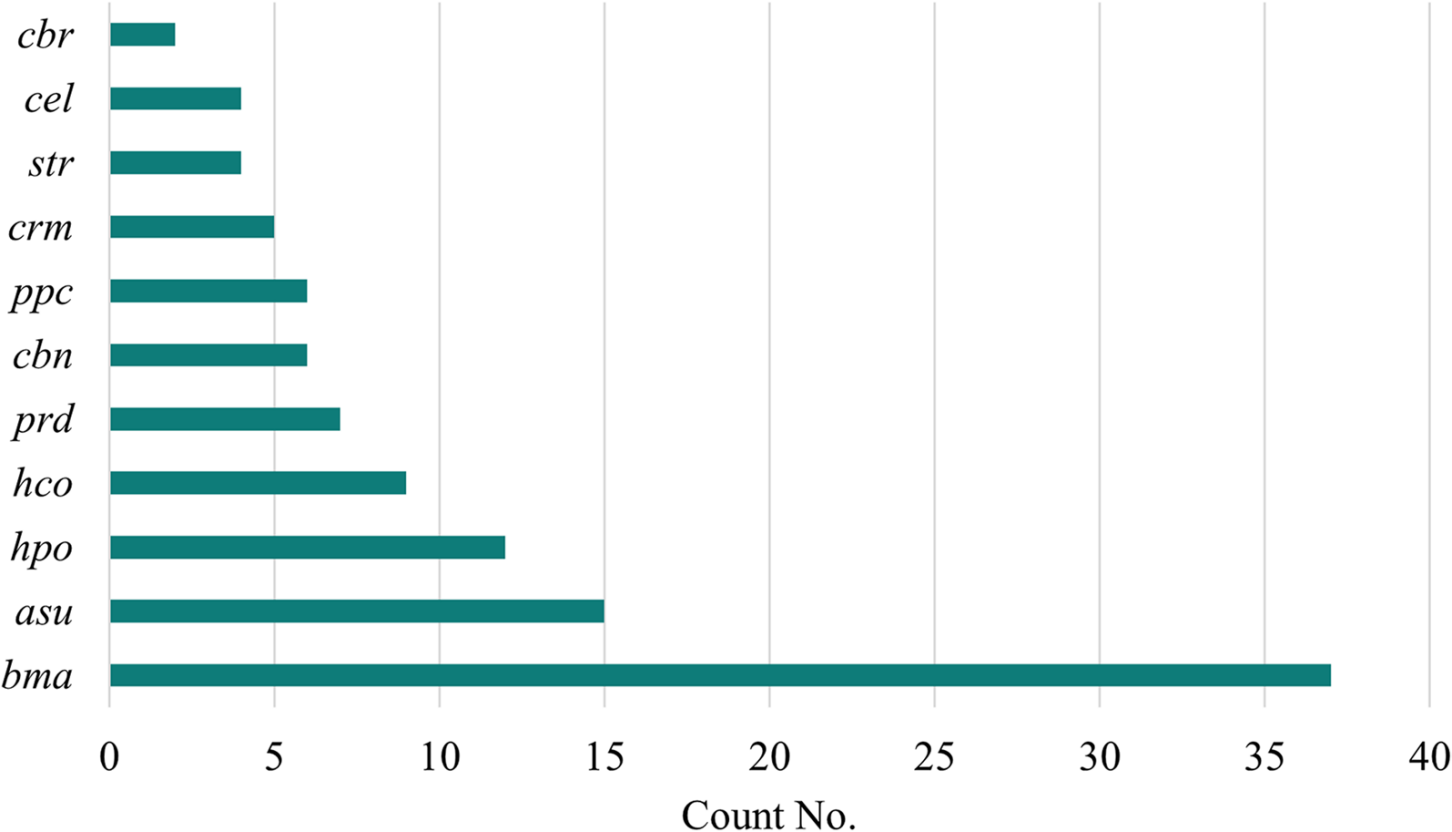
Conservation profile of the identified miRNAs. The count number of different conserved miRNAs with nematode species found in miRbase 22. *cbr* *Caenorhabditis briggsae*, *cel* *Caenorhabditis elegans*, *str* *Strongyloides ratti*, *crm* *Caenorhabditis remanei*, *ppc* *Pristionchus pacificus*, *cbn* *Caenorhabditis brenneri*, *prd* *Panagrellus redivivus*, *hco* *Haemonchus contortus*, *hpo* *Heligmosomoides polygyrus*, *asu* *Ascaris suum*, *bma* *Brugia malayi*

After RNA extraction and sequencing of each sample, the total raw reads from M0-7, I0-7, and J0-7 were 265,907,220. These sequences underwent rigorous filtering, which included the removal of junk reads, wrong-size sequences, and low-quality sequences, resulting in changing the number of valid reads to 241,568,053 (90.84%) (Table 2). A total of 66 sequence candidates were present at ≥ 10 copies after normalization (Supplementary Table S1). Only three of those miRNA candidates have a high expression level, meaning the number of reads was higher than the average count of the dataset. These three candidates were bma-let-7_R-1, bma-miR-71_R + 2, and bma-miR-92_R + 1_1ss10CT (Table 3). The miRNAs bma-let-7_R-1 and bma-miR-92_R + 1_1ss10CT were found in all the sample timepoints (M0-7, I0-7, and J0-7). However, bma-miR-71_R + 2 was restricted to M6-7, I7, and J7. The remaining 63 sequence candidates were below the average number but with ˃ 10 copies and we identified the 7 miRNA sequences with the higher expression level (higher number of reads after normalization) among this group (Table 3). A total of five of the candidates within this group were consistently expressed in all sample timepoints (M0-7, I0-7, and J0-7). Those were bma-miR-7_1ss10TA, bma-miR-100b_R-1_1ss16AT, asu-miR-1-3p_R-1, asu-let-7-5p_R + 1, and cbr-let-7_R + 1_1ss12GA. Only two candidates of this group were expressed after 28 weeks post-infection (bma-miR-100d_R + 1 and bma-miR-81_R + 1). A total of 37 candidate sequences were found < 10 copies after normalization; usually found in M7, I7, or J7 timepoints (28 weeks after infection) (Supplementary Table S1). The samples from 28 weeks after infection had a higher number of different expressed miRNA candidate sequences, M7 (70), J7 (39), and I7 (62) (Table 4). Of the 103 miRNA sequence candidates, 42 have no known nematode homologs (group gp4), thus considered potential candidates of unique *D. immitis* sequences. The remaining 61 represented a conservation profile of 11 nematode species (Fig. 1). The target gene prediction analysis using the GO and KEGG enrichment analysis of target genes demonstrated that the miRNA candidate genes are involved in several metabolic pathways, cellular processes including DNA reparation, protein processing in endoplasmic reticulum, and signaling pathways including mitogen-activated protein (MAP) kinase cascade (Supplementary Tables S2, S3, S4).

**Table 2:** Overview of reads from raw data to cleaned sequences for miRNA identification

Sample ID	Total reads	Valid reads	% of total reads
M0	9,742,097	8,152,273	83.68
M1	19,349,725	18,779,726	97.05
M2	11,444,206	10,514,070	91.87
M3	8,942,072	8,528,578	95.38
M4	10,836,840	9,608,572	88.67
M5	8,438,990	8,078,546	95.73
M6	11,266,954	9,765,282	86.67
M7	9,680,997	6,356,458	65.66
I0	10,128,774	9,782,532	96.58
I1	9,601,775	9,290,260	96.76
I2	12,444,397	9,454,368	75.97
I3	17,346,706	17,009,625	98.06
I4	10,578,368	10,407,057	98.38
I5	11,633,490	11,091,242	95.34
I6	8,617,848	8,244,683	95.67
I7	10,602,694	9,303,351	87.75
J0	11,399,620	10,598,158	92.97
J1	12,633,418	11,484,146	90.90
J2	13,478,260	12,104,999	89.81
J3	10,812,202	10,547,224	97.55
J4	8,152,329	7,502,555	92.03
J5	9,325,631	8,293,317	88.93
J6	9,561,594	8,484,195	88.73
J7	9,888,233	8,186,836	82.79
Total reads	265,907,220	241,568,053	90.84

**Table 3:** List of the ten most abundant miRNA candidates identified in three dogs experimentally infected with *Dirofilaria immitis* (M, I, J) at all timepoints

miRNA ID	Sequence	Previous studies	Mapped to dog miRbase 22.0
bma-let-7_R-1	TGAGGTAGTAGGTTGTATAGT	[11–13, 31] (MYL3-4)***	cfa-let-7a
bma-miR-92_R + 1_1ss10CT	TATTGCACTTGTCCCGGCCTG	–	cfa-miR-92a
bma-miR-71_R + 2*	TGAAAGACATGGGTAGTGAGA	[11–13, 31] (MYL3-4)***	–
bma-miR-7_1ss10TA	TGGAAGACTAGTGATTTTGTTGT	[13, 31] (MYL3-4)***	cfa-miR-7
bma-miR-100b_R-1_1ss16AT	AACCCGTAGATCCGATCTTGT	[31]	cfa-miR-99a
bma-miR-81_R + 1*	TGAGATCATTGTGAAAGCTATT	[12, 13, 31] (MYL3-4)***	–
bma-miR-100d_R + 1**	TACCCGTAGCTCCGAATATGTGT	[11–13, 31] (MYL3-4)***	cfa-miR-10b
asu-miR-1-3p_R-1	TGGAATGTAAAGAAGTATGT	[11–13] (MYL3-4)***	cfa-mir-1–2
asu-let-7-5p_R + 1	TGAGGTAGTAGGTTGTATAGTTT	[11–13] (MYL3-4)***	cfa-let-7a
cbr-let-7_R + 1_1ss12GA	TGAGGTAGTAGATTGTATAGTTT	[12]	cfa-let-7f

^*^Only expressed in M6-7, I7, and J7. **Only expressed in 28 weeks post-infection (M7, I7, and J7). ***Missouri and Yazoo strains L3 and L4

**Table 4:** The number of miRNA candidates differentially expressed in three infected dogs at eight different timepoints

Sample ID	Weeks post-infection	Subject			
		M	I	J	M,I,J**
0	0*	18	9	13	7
1	4	10	6	10	6
2	8	10	23	11	9
3	12	7	6	6	6
4	16	10	6	7	6
5	20	7	7	10	7
6	24	13	7	10	7
7	28	70	62	39	29

*Blood samples taken 1 day prior to subcutaneous inguinal inoculation with L3 *Dirofilaria immitis*. These samples are considered negative controls. **A total of 5 miRNA candidate sequences were present at all sample timepoints (M0-7, I0-7, J0-7)

## Discussion

The diagnostic techniques (antigen, microfilaria, and PCR) used in tandem aligned with the detailed description of the HW life cycle in dogs (Table 1). The life cycle was completed in 7 months in this study, like the literature that reports 7–9 months [1, 3, 21, 22].

In this project, HW remained undetectable until 20 weeks after infection, approximately 5 months or 140 days post-infection. The antigen testing after heat treatment was the first ancillary test able to detect HW. The use of heat treatment as an immune complex dissociation (ICD) technique enhanced the antigen detection in experimental assays and surveys [23–33]. In our experimental design, we justified the use of heat treatment before antigen testing on the basis of a previous study that reported that the use of heat treatment before antigen testing allowed for HW detection as early as 98 days post-infection (mean 126.9 days, SD ± 18.9 days using DiroCHEK^®^) in experimentally infected dogs [30]. In the same study without the heat treatment before antigen testing, HW was diagnosed as early as 140 days (mean 162.6 days, SD ± 23.0 days using DiroCHEK^®^) [30]. As suggested that heat treatment may lead to false negative results [25, 35, 36], ideally the antigen detection assay should have been performed before the heat treatment in this work.

Our results demonstrate that L3, fourth-stage larvae (L4), and juvenile adult worms are still undetectable by the diagnostic methods during the early stages of infection, before the worms reaching sexual maturity. Microfilariae are present within the blood of infected dogs by 180–210 days post-infection, completing the cycle [3, 17]. PCR detected nucleic acids derived from *D. immitis* 24 weeks, 168 days, or 6 months post-infection. Microfilaria visualization was achieved in the three subjects after 28 weeks (196 days, 7 months post-infection). Our results reflect that PCR was more sensitive in detecting circulating microfilaria than the Giemsa-stained thick smear. However, PCR was unable to detect the early developmental stages of HW. We speculate that PCR could potentially detect fragments of sloughed cuticles from adult worms and other developmental stages and remnants of dead worms. However, on the basis of the timeline of our results, we strongly suggest that microfilaria is the main source of the DNA amplified by the PCR assay.

The search for miRNAs as potential detection biomarkers of early HW infection has been described through several publications in the last 10 years [11, 16–18, 35]. The utilization of miRNA deep sequencing and bioinformatic analysis in our study identified potential candidate *D. immitis* miRNA sequences in all of the blood samples (*n* = 24) obtained from the three subjects (I, J, M) at all infection timepoints (4–28 weeks post-infection) and the controls. While previous studies have successfully obtained possible candidate *D. immitis* miRNAs directly from adult worms after maceration and RNA extraction, or from culture media containing various developmental stages, including adult males and females and L4, L3, and microfilariae, the technical intricacies of these experiments and the sample specificity raise questions about their relevance to host conditions in clinical infections [16, 17, 25].

An ideal miRNA candidate sequence should be detected consistently in the blood with a high-to-middle expression level and be specific in determining HW infection. Our experimental design demonstrated that HW-derived miRNA sequence candidates are expressed in blood during the first 28 weeks post-experimental infection. To use a miRNA sequence candidate as a biomarker of early disease, it should be consistently detected in all or the majority of the timepoints except the control, determining the presence of early developmental stages of *D. immitis*. Regrettably, none of the miRNA candidate sequences identified in this study fulfilled these criteria. Notably, 7/10 of the most abundant miRNA candidate sequences were consistently detected at high (*n* = 2) or middle (*n* = 7) expression levels in all the timepoints, including the control (Supplementary Table 1). Interestingly, six of these miRNA candidate sequences were previously described in experimental studies, as circulating from chronic HW infections, macerated adult worms, microfilariae, and L3 and L4 stages from Yazoo (resistant to macrocyclic lactones) and Missouri (susceptible to macrocyclic lactones) strains [11, 16, 17, 36] (Table 3). Only one miRNA candidate was not previously described (bma-miR-92_R + 1_1ss10CT). A strength of our experiment lies in the use of blood samples from animals with a well-defined infection timeline and the known number of L3 inoculated in each animal. We included a sample taking the previous inoculation that served as an “internal negative control.” The fact that these seven miRNA candidate sequences were found at all sample points indicates that such sequences represent a conserved miRNA. We were able to map all these sequences to the *Canis lupus familiaris* database at miRBase 22.0 (Table 3). This indicated the selected miRNA sequence candidates are conserved sequences among different species and not specifically derived from *D. immitis.* Alternatively, such miRNA candidate sequences could be derived from other nematodes; however, the three dogs in this study were maintained at the University of Georgia under strict experimental conditions and veterinary care, thus an undiagnosed nematode co-infection is extremely unlikely. Thus, we do not interpret these findings as possible early biomarkers of disease.

Three additional miRNA candidate sequences were found at high and middle expression levels, with a more restricted distribution. Such miRNA candidates’ sequences were bma-miR-71_R + 2 and bma-miR-81_R + 1, found at 28 weeks post-infection in the three subjects (M, I, J) and at 24 weeks only in subject M. The other miRNA candidate sequence was bma-miR-100d_R + 1, detected only at 28 weeks after infection in the three subjects (Table 3). Additionally, bma-miR-71_R + 2 and bma-miR-81_R + 1 were not able to be mapped on *C*. *familiaris* miRBase 22.0, suggesting its specificity and promising candidates to use in diagnosis. Both sequences were previously described in L3, L4, adults, and microfilaria from *D. immitis* Missouri and Yazoo strains [11, 16, 17, 36]. We speculate that its presence at this stage of infection is derived from adult worms or microfilariae. The importance of unique *D. immitis*-derived miRNA sequence candidates relies on their reliability for diagnostic purposes, in which such sequences are not derived from other nematodes, precluding their use. In addition to canine HW, the sequences could be tried to be explored in feline HW diagnoses, since the published study neglected its use in cats [15].

We accurately determined that most of circulating *D. immitis*-derived miRNAs are detected after 28 weeks of infection (Supplementary Table 1; Table 4). Before 28 weeks, the expression levels are erratic and not consistent in the majority of the candidates. While circulating miRNAs derived from HW may be detectable in chronic infections [15], our analysis suggests that they cannot reliably detect infection before 28 weeks post-infection. Most of the identified miRNA sequence candidates were expressed after 28 weeks. A previous study used two miRNA candidate sequences (miR34 and miR-71) as potential molecular markers of infection in chronically infected dogs [18]. The authors conclude that both candidates worked as biomarkers for the presence of *D. immitis* but cannot predict the parasite burden of the adult worms [18]. Our results were in agreement with previous studies that miRNA detection could be sensitive methods but not specific for HW diagnosis. Consequently, such assays may be impractical and unnecessary for veterinary practitioners, given the speed, ease, and reliability of antigen testing for early diagnosis in clinical settings. However, they could represent an alternative diagnostic method for chronic infections.

A total of 42 potential *D*. *immitis* unique candidates were identified, however; 34 candidates were expressed only 28 weeks after infection in one of the three subjects, with 25 having low levels of expression (Supplementary Table 1). In subject I, 5 miRNA unique potential candidates were expressed at low levels 8 weeks (I2) post-infection (PC-5p-216952_11, PC-5p-104864_25, PC-3p-180647_14, PC-5p-114309_23, PC-3p-34927_64) and became undetectable thereafter. PC-3p-34927_64 had a low expression level in subject J 20 weeks after infection (J5); however, it became undetectable thereafter. In subject M, none of the potential *D. immitis* unique candidates was detectable before 28 weeks post-infection (M7). It is important to consider that a number of the predicted candidates might represent artifacts and not true miRNA sequences [17]. The identification of potential target genes in this study yielded several mechanisms and cellular processes affected by the miRNA (Supplementary Tables 2, 3, 4) and through this analysis, the miRNA regulatory network can be characterized to better describe its relevance to putative target transcripts [36].

A notable study by Tritten et al. [15] successfully reported the identification of circulating miRNAs from HW-infected dogs. The authors demonstrated the presence of *D. immitis* miRNAs through real-time quantitative PCR on blood samples from infected dogs. However, several differences between their study and ours may explain the variance in results. Notably, Tritten et al. [15] detected more than 200 *D. immitis*-derived possible candidate miRNAs in plasma, while we performed our analysis using whole blood. Our approach was based on previous studies indicating that miRNAs remain stable in whole blood compared with plasma or serum [36]. Despite efforts to maintain sample integrity during transportation and processing in this study, unexpected ribosomal ratios and partially degraded RNA were observed during quality control analysis of the RNA library. Nonetheless, the samples were deemed suitable for miRNA sequencing, given the stability of small RNA compared with larger RNA molecules such as ribosomal RNA.

A limitation of our study was the small number of subjects (*n* = 3). In contrast, Tritten et al. [15] utilized five dogs experimentally infected with *D. immitis*, extracting higher volumes of plasma (7.5–10 mL per animal after drawing 20 mL of whole blood). Additionally, they submitted a pool of total RNA from four experimentally infected dogs and an additional sample from naturally HW-infected dogs co-infected with hookworms for miRNA deep sequencing and bioinformatic analysis. In our study, individual analysis was performed without pooling samples. Furthermore, the previous study did not specify the age of infection for each dog, precluding definitive conclusions regarding the use of candidate miRNAs as biomarkers for early diagnosis [34].

## Conclusions

Our study sheds light on the diagnostic challenges associated with the detection of early developmental stages of HW infection in dogs and the efficacy of different diagnostic methods. Our findings indicate that early developmental stages and juvenile adult worms cannot be reliably detected using miRNA deep sequencing and bioinformatic analysis until 28 weeks of infection. However, circulating miRNA can be used to diagnose chronic infections. Antigen detection after the ICD heating method is the most sensitive method for early diagnosis of HW, demonstrating superior sensitivity compared with other methods evaluated in this study. However, ICD is not performed in veterinary clinics. PCR emerges as a more sensitive method for microfilariae detection when compared with Giemsa-stained thick smear. Continued research and evaluation of diagnostic techniques are essential for improving the timely detection and management of HW infection, ultimately contributing to better outcomes for affected animals.

### Supplementary Information

Supplementary Material 1.

Supplementary Material 2.

Supplementary Material 3.

Supplementary Material 4.

## Data Availability

All data supporting the conclusions of this study can be found in the manuscript and supplementary files.

## Abbreviations

BHQ2: Black hole quencher 2
EDTA: Ethylenediaminetetraacetic acid
ELISA: Enzyme-linked immunosorbent assay
HW: Heartworm
HWD: Heartworm disease
ICD: Immune complex dissociation
miRNA: Micro RNA
ROX: 6-Carboxy-Xrhodamine
TAE: Tris–acetate-EDTA
